# The impact of greening interventions in school grounds on social behavior and cognitive performance among primary school children

**DOI:** 10.3389/fpubh.2025.1620199

**Published:** 2025-10-29

**Authors:** Lore Verheyen, Bo H. W. van Engelen, Bjorn Winkens, Kenneth Vanbrabant, Ellen Hannes, Tim S. Nawrot, Robert Malina, Onno C. P. van Schayck, Michelle Plusquin

**Affiliations:** ^1^Environmental Biology, Centre for Environmental Sciences (CMK), Hasselt University, Diepenbeek, Belgium; ^2^Department of Family Medicine, Care and Public Health Research Institute (CAPHRI), Maastricht University, Maastricht, Netherlands; ^3^Department of Methodology and Statistics, Care and Public Health Research Institute (CAPHRI), Maastricht University, Maastricht, Netherlands; ^4^Environmental Economics, Centre for Environmental Sciences (CMK), Hasselt University, Diepenbeek, Belgium

**Keywords:** school greening, cognitive performance, socio-emotional wellbeing, biodiversity, eye-tracking

## Abstract

**Background:**

Green spaces have been identified as beneficial for children’s mental health, as well as cognitive performance, however, less is known about the role of biodiversity within these environments. Here, we study the impact of greening schoolyards, especially with regard to biodiversity enrichment, on children’s behavior, emotion recognition, cognitive performance and mental effort, and attentional bias, compared to observations in control schools.

**Methods:**

This study employs an interventional design, including two intervention and two control schools located in Belgium and the Netherlands. Data was collected from 169 children aged 7–12 years, assessing cognitive performance using a battery of cognitive tasks combined with eye tracking, as well as socio-emotional wellbeing via eye tracking and validated questionnaires. Baseline measurements were followed up every 6 months for 2 years.

**Findings:**

Selective attention in the Stroop test and mental effort, as indicated by enlarged pupil diameters during cognitive tasks, increased more over time in the intervention group compared to the control group as schoolyard greening progressed. The intervention was associated with improved scores on prosocial behavior over time. These associations were independent of sex, age, country, education level of the household, the capacity to manage household incomes, and season. Attentional bias towards the emotion of happiness using eye-tracking did not show a significant difference in changes over time between intervention and control groups.

**Interpretation:**

These findings suggest that a green, more biodiverse school environment could improve children’s cognitive and emotional functioning, highlighting the importance of designing schoolyard green spaces that enhance both nature contact and biodiversity as a valuable public health initiative.

## 1 Introduction

Mental health issues and cognitive development concerns in children are growing global challenges with wide-ranging implications for education and long-term wellbeing due to educational attainment and higher dropout rates (1–3). These issues might affect education and overall health, potentially limiting adolescents’ ability to attain higher levels of education, long-term professional opportunities, and socio-economic stability (2, 3). UNICEF data reveals that 86 million adolescents aged 15–19 years and 80 million children aged 10–14 years are facing mental disorders globally (4).

Spending time in green environments has been shown to positively influence physical and mental health and improve outcomes across various cognitive and emotional domains (5–10). Children exposed to nature are less likely to develop psychiatric disorders later in life (11), and research increasingly supports the hypothesis that natural spaces can enhance cognitive functions, including attention and memory (12–14). The attention restoration theory (ART) and stress recovery theory (SRT) provide a conceptual framework for these benefits, suggesting that natural environments help restore depleted attention capacities (15, 16) and reduce physiological stress responses (17).

Greening schoolyards not only has the potential to enhance cognitive performance and emotional health but also offers a unique opportunity to promote environmental education and greater engagement with nature throughout life (18–20). However, access to green space in urban areas for young people is changing as urbanicity is increasing (21). Increasing urbanization and densification often reduce opportunities for everyday contact with nature, contributing to the so-called ‘Extinction of experience’ or ‘Nature-deficit disorder’ (20–22). School environments are particularly well-suited to address this disconnection to nature by introducing green schoolyard interventions, providing structured, daily, and meaningful interactions with green spaces, as children spend a significant portion of their day at school.

In urban areas, green spaces are not only important for human health but are also linked to maintaining biodiversity (23–27). Biodiversity-rich environments provide more varied sensory experiences, which can enhance cognitive performance, general health, and wellbeing (25, 28–30), and play a key role in species conservation by creating habitats for wildlife such as birds and insects (26, 31). Furthermore, urban greening and school greening projects can contribute to moral development, which encourages children to care for and respect nature more, and raise awareness about biodiversity loss (32–34). Integrating greening initiatives in educational programs can thus benefit both children and the broader urban environmental ecosystem.

Despite promising theories and findings, there is limited experimental research specifically targeting the impact of schoolyard greening and biodiversity enrichment on children’s cognitive and emotional wellbeing (35–37). Further research is therefore needed to address gaps in understanding the role of greening initiatives that enhance biodiversity in schoolyards, providing insights into their long-term outcomes and ensuring their overall effectiveness.

This study investigates the potentially beneficial effects of greening schoolyards on children’s cognitive performance and socio-emotional wellbeing by addressing four key questions: First, we investigate whether greening schoolyards improves children’s cognitive performance and mental engagement over time. We hypothesized that the children involved in the intervention school will show faster reaction times and greater pupil dilation during cognitive tasks compared to controls. Second, we examine whether greening influences children’s visual attention toward positive emotional stimuli. We hypothesize that the intervention group will display an increased initial fixation duration ratio toward happy versus negative facial expressions. Third, we investigate whether schoolyard greening changes children’s visual preference for emotions. We hypothesize that the intervention group will allocate a greater proportion of first fixation duration to the eye region of faces. Fourth, we evaluate whether greening improves children’s prosocial behavior. We hypothesize that children from the intervention schools will report higher prosocial behavior scores. By focusing on these aspects, this research highlights the potential of green schoolyards to support children’s health and cognitive development from a young age.

## 2 Methods

This explorative intervention study, with a 2-year follow-up, investigates the impact of greening schoolyards on cognitive performance and socio-emotional wellbeing. This study was registered at ClinicalTrials.gov (NCT04898439) and approved by the Commission for Medical Ethics of Hasselt University (CME2021/042) and is a part of the original ‘Healthy Primary School of the Future’ trial (METC-Z no. 14-N-142).

### 2.1 Study design

This study employed a non-randomized controlled trial involving four primary schools, with one intervention and one control school located in Limburg, Belgium, and the other intervention and control school located in Limburg, the Netherlands. All participating schools were located in urban areas according to the urban–rural classification system (38), which defines urban areas as those with a higher population density and built-up environment. Estimated schoolyard area per child and percentage schoolyard greening within this study per school are provided in Supplementary Table 1. We carried out interventions involving creating designated play areas aimed at increasing biodiversity through the incorporation of height variation and structural diversity, using native plants, and providing varied vegetation structure. This initiative not only aimed at increasing vegetation but also aimed at supporting greater species diversity, contributing to a richer ecological setting. The green schoolyards were designed to create interactive spaces where children could engage with nature, encouraging both free play and structured outdoor activities. The control schools maintained their usual paved playgrounds with minimal vegetation during the study period. Randomization was not possible due to the necessity for complete voluntary cooperation and participation of the intervention schools in greening the schoolyard.

### 2.2 Data collection

Data were collected at five time points: the baseline assessment took place in November 2021, and subsequent assessments in April 2022 (except for the Dutch schools), November 2022, April 2023, and November 2023 (except for the Dutch schools) (Supplementary Table 2). The school management was personally informed about the importance, purpose, design, and possible risks of the study. After permission from the management, the children and parents were informed by a class visit for the children and a short info flyer with an attached comprehensive information letter for the parents. Before the baseline examination, written consent was obtained from the parents, and the children gave their oral permission to participate. The information letter and related materials were provided in Dutch. Copies of these documents are available from the authors upon request.

### 2.3 Outcomes

Data collection during all assessment moments included several self-reported questionnaires to assess socio-emotional wellbeing, and cognitive performance assessment in combination with eye-tracking to assess attentional bias. An overview of all outcome measures collected at each assessment is given in Supplementary Table 2. A general questionnaire regarding information about parental education, parity, ethnicity, parental occupation, income, household smoking behavior, illness, socio-economic demographics, the birth date of the child, the sex of the child, and the use of medication was filled out by a parent at home before the baseline examination. These variables were selected *a priori* on the basis of background knowledge about the relationship of the variable to the outcomes. Completion of privacy-sensitive questions, such as parental body mass index (BMI), socioeconomic status (SES), and disease status, was optional.

### 2.4 Cognitive performance and mental effort using eye-tracking

Cognitive functioning was assessed using a battery of five consecutive cognitive tests on a computer, guided by a trained staff member using the mental information processing and neuropsychological diagnostic system (MINDS) (39). Cognitive performance assessments were conducted individually in a separate room during regular class hours. At least one researcher was present to supervise them and address any questions. The task order was fixed for all participants during all assessments and took approximately 30 min. The cognitive battery consisted of five tasks. The continuous performance test (CPT) was administered to evaluate attention and concentration (40–42). In this task, a series of stimuli were sequentially displayed on a screen, and participants were required to respond (using a space bar, mouse click, touchscreen, or external button box) only when a specific target combination of two stimuli appeared (e.g., the letter X following the letter A). The frequency and type of errors made during the task provided insights into potential impulsivity or attention deficits. The average reaction time of correct responses (ms) was used as the outcome variable. The second assessment, the symbol digit modalities test (SDMT), measured information processing speed (42). Participants used a coding key of nine abstract symbols, each paired with a number, to quickly identify and match numbers corresponding to the presented symbols. The average time per response (seconds) is used as the outcome measure. The third test, the SPANNE test, assessed short-term memory (42). During this task, participants were instructed to recall and reproduce sequences of numbers both in the forward and backward directions. In the analyses, the maximum number of digits recalled was used as the outcome measure. The fourth test, the signal detection test (SDT), evaluated the visual information processing speed. Participants were tasked with identifying whether a deviant symbol was present within a series of visual stimuli as quickly as possible (42, 43). In the analyses, the average reaction time (ms) for correct responses is presented. Lastly, the fifth test, the STROOP task, assessed selective attention (42, 44, 45). Participants were presented with a color word displayed in an incongruent color (e.g., the word “red” printed in blue) and instructed to identify the color of the text rather than the word itself. For the last test, the average reaction time after log transformation was used as the analysis outcome. To assess mental effort, the Tobii Pro-Nano eye-tracking device (Tobii, Stockholm, Sweden) was used to determine the pupil dilation of the participants during all tests (46). The eye-tracking device was placed on the bottom of the screen of the tablets on which the tests were performed. Prior to beginning the eye-tracking session, a calibration process was conducted as recommended by the software. Using the Tobii Pro lab software (Version 24.21), the pupil diameter was calculated during each task. Pupil dilation can be used as a biomarker of effort in cognitive tasks (46).

### 2.5 Attentional bias using eye-tracking

Attentional bias was assessed using a Tobii Pro eye-tracking device while participants viewed emotional faces, following procedures similar to those described by Goeleven et al. (47). Attentional bias assessment was conducted individually in a separate room during regular class hours directly after the battery of cognitive tasks. Areas of interest (AOIs) were defined within the Tobii Pro lab software for faces depicting happy, anxious, and angry expressions (Supplementary material 1). Eye movement data (first fixation duration in milliseconds) were then used to calculate the ratio of attention directed toward the happy AOI relative to the anxious and angry AOIs. Specifically, a higher ratio for happy versus anxious/angry AOIs suggests a stronger initial preference or bias toward positive stimuli (i.e., a positive attentional bias) (48, 49). Conversely, a lower ratio may indicate that attention is preferentially captured by negative expressions, consistent with a negative attentional bias. This ratio-based approach for measuring attentional bias is grounded in findings that individuals with specific emotional or clinical traits (e.g., anxiety, depression) often show systematic differences in how they orient and maintain their gaze on emotional stimuli (49, 50). By comparing the duration of the first fixation on happy AOIs with those on anxious and angry AOIs, we gain insight into whether participants’ attention is initially guided by positive or negative emotional cues.

### 2.6 Emotion recognition

Visual preference was assessed using the same emotional faces as during the attentional bias test. Eye movement data (first fixation duration in milliseconds) were used to calculate the proportion of first fixation duration specifically allocated to the eye region compared with the forehead and mouth. The eye region is often considered the primary region for emotion recognition and can be particularly important in detecting social cues (51). A visual example illustrating how the AOIs were defined in Tobii Pro software can be found in Supplementary material 1.

### 2.7 Behavioral assessment

During each timepoint, the child’s behavior was evaluated using the Strengths and Difficulties Questionnaire (SDQ) filled out by a parent (44, 52, 53). Questionnaires were sent to the parents via mail to be completed at home. This publicly available, validated tool measures the emotional and behavioral wellbeing of children and can be found online (54). The SDQ consists of five subscales: emotional problems, conduct problems, hyperactivity/inattention, peer relationship problems, and prosocial behavior. Responses are rated on a three-point scale (0, 1, 2), and a total difficulty score is calculated by summing the scores from the first four subscales (emotional symptoms, conduct problems, hyperactivity/inattention, and peer relationship problems). A higher total difficulty score reflects more risk of abnormal behavior, whereas for prosocial behavior a lower score indicates more risk of experiencing abnormal behavior.

### 2.8 Statistical analysis

Data were analyzed using RStudio software, version 4.4.0 (RStudio Inc., Boston, USA). Continuous data are presented as means and standard deviations (SD), and categorical data are presented as numbers and frequencies (%). Due to the explorative nature of this study, no sample size calculations were performed. Data normality was checked using the Shapiro–Wilk test. Baseline differences in demographic characteristics between the control and intervention groups were assessed using independent samples *t*-test for continuous variables and Chi-Squared tests for categorical variables. These preliminary tests were used solely to check for potential systematic bias at baseline and were not part of the main outcome analyses. All four research questions were analyzed using linear mixed-effects regression models, accounting for fixed and random effects. Fixed effects included group (intervention vs. control) and examination number (baseline, after 6 months, after 12 months, after 18 months, and after 24 months), while random intercepts accounted for individual variability among participants, *a priori* chosen covariates included age (years), sex, country in which the school is located, education level of the household (low, middle, or high when parents indicated their highest education level as ‘no diploma or primary school’, ‘secondary school’, or ‘college of university’, respectively), the capacity to manage household incomes (very difficult, difficult, average, rather easy, or very easy), and the season at the moment of the examination (spring and summer were coded as warm season, and winter and fall were coded as cold season). An interaction term for the examination (consecutive moment of examination) and group (control group vs. intervention group) was included to assess differences over time per treatment group. School identity was not included as a separate random factor, as the combination of country and treatment group already accounts for this structure. This method provides an indication of the effect of the intervention when the intervention is progressing. The assumption of linearity was assessed using residual plots. Akaike information criterion (AIC) and Bayesian information criterion (BIC) were used for model selection after the addition of a quadratic term to the model when patterns indicative of non-linearity were observed. All pre-specified covariates and interaction terms were retained in the final models regardless of AIC values. Selection bias was assessed for all outcomes by checking for baseline differences between the intervention and control schools using an independent samples *t*-test (Supplementary Table 3). Beta estimates, 95% confidence intervals, and *p*-values were provided for key outcomes. Beta estimates are abbreviated as beta (*β*) and can be interpreted as a change in outcome having the green school intervention compared to the control schools over time. Statistical significance was set at *p* ≤ 0.05. Estimated means and standard deviations for each examination in comparison to the baseline measurement are provided in Supplementary Table 4.

### 2.9 Population characteristics

Out of 482 students, a total of 169 children aged 7–12 participated in the study of which 86 in the intervention group and 83 in the control group (Supplementary Figure 1). The participation rate was 44.1% and 28.9% for the intervention and control schools, respectively. Demographic characteristics did not differ significantly between the intervention and control schools (Table 1). The average age of the participating children was 10 years. The majority of the children were girls (55% vs. 45%). The mean BMI *z*-score was 0.40. Approximately one-third of mothers (36.1%) had attained a medium level of education, defined as completing secondary school. A substantial proportion of fathers (39.1%) are highly educated; holding a college or university degree. Parents most commonly reported that their household income allows them to get by without significant difficulty but also without ease (28.4%).

**Table 1:** Baseline characteristics of participating children in intervention and control schools, including test statistics, degrees of freedom, and *p*-values for group comparisons.

Characteristics	Control schools (*n* = 83)	Intervention schools (*n* = 86)	Test statistic (df)	*p*-value
	Mean ± SD or *n* (%)	Mean ± SD or *n* (%)		
Age, years	10.2 (1.1)	10.0 (1.0)	*t* = 1.15 (164.6)	0.25
Sex			*χ*^2^ = 0.13 (1)	0.71
Boy	39 (47.0%)	37 (43.0%)		
Girl	44 (53.0%)	49 (57.0%)		
BMI *z*-score	0.52 (1.14)	0.294 (1.16)	*χ*^2^ = 1.21 (154)	0.23
Education mother			*χ*^2^ = 1.93 (2)	0.38
Low	14 (16.9%)	20 (23.3%)		
Middle	34 (41.0%)	27 (31.4%)		
High	27 (32.5%)	24 (27.9%)		
Missing	8 (9.6%)	15 (17.4%)		
Education father			*χ*^2^ = 5.23 (2)	0.07
Low	2 (2.4%)	9 (10.5%)		
Middle	35 (42.2%)	28 (32.6%)		
High	33 (39.8%)	33 (38.4%)		
Missing	13 (15.7%)	16 (18.6%)		
Income			*χ*^2^ = 2.55 (4)	0.64
Very difficult	1 (1.2%)	2 (2.3%)		
Difficult	3 (3.6%)	4 (4.7%)		
Average	27 (32.5%)	21 (24.4%)		
Rather easy	22 (26.5%)	11 (12.8%)		
Very easy	14 (16.9%)	9 (10.5%)		
Missing	16 (19.3%)	39 (45.3%)		

This study employed a non-randomized controlled trial involving four primary schools, with one intervention and one control school located in Limburg, Belgium, and the other intervention and control schools located in Limburg, the Netherlands. General characteristics were collected regarding information about the birth date of the child, the sex of the child, parental education, and income using a questionnaire before the baseline examination.

Values are presented as mean ± standard deviation (SD) for continuous variables and *n* (%) for categorical variables. Group comparisons for continuous variables were conducted using Welch’s two-sample *t*-test; categorical variables were compared using Pearson’s Chi-squared test. The *p*-value for the difference between groups is reported.

df, degrees of freedom. *p*-values < 0.05 are considered statistically significant. BMI, body mass index.

## 3 Results

### 3.1 The effect of schoolyard greening on cognitive performance and pupil diameter

The intervention group showed more increased selective attention and larger pupil diameter in multiple cognitive tests during greening as compared to the control group. An inverse significant association was found for the interaction effect between intervention and control group throughout greening on selective attention during the stroop test (*β* = −0.01, 95% CI −0.03 to −0.002, *p* = 0.02) after adjustment for age, sex, country in which the school is located, education level of the household, the capacity to manage household incomes, and season. No significant interaction effects were found for the outcomes of the continuous performance test, signal detection modalities test, spanne test, and signal detection test during greening. An interaction effect between intervention and control group throughout greening was observed for pupil diameter during the signal detection modalities test (*β* = 0.09, 95% CI −0.01 to 0.19, *p* = 0.08), spanne test (*β* = 0.11, 95% CI 0.02–0.20, *p* = 0.01), signal detection test (*β* = 0.11, 95% CI 0.01–0.21, *p* = 0.03), and stroop test (*β* = 0.08, 95% CI −0.02 to 0.17, *p* = 0.1) independent of age, sex, country in which the school is located, education level of the household, the capacity to manage household incomes, and season. An overview of estimates is provided in Table 2.

**Table 2:** Estimates, confidence intervals, and *p*-values for the interaction effects between intervention and control group throughout greening on cognitive tests and eye-tracking analysis regarding pupil diameter.

Outcome	*β*	2.5% CI	97.5% CI	*p*-value
Cognitive performance				
CPT	−1.52	−13.91	10.87	0.81
SDMT	−0.03	−0.16	0.11	0.70
SPANNE forward	−0.03	−0.17	0.10	0.62
SPANNE backward	−0.03	−0.18	0.11	0.64
SDT	−42.31	−99.05	14.44	0.14
STROOP	−0.01	−0.03	−0.002	**0.02**
Pupil diameter				
CPT	0.04	−0.04	0.13	0.34
SDMT	0.09	−0.01	0.19	0.08
SPANNE	0.11	0.02	0.20	**0.01**
SDT	0.11	0.01	0.21	**0.03**
STROOP	0.08	−0.02	0.17	0.10

The model was adjusted for age, sex, country, education level of the household, the capacity to manage household incomes, and season.

Beta estimates are abbreviated as beta (*β*) and can be interpreted as a change in outcome having the green school intervention compared to the control schools over time. Bold indicates a significant *p*-value (< 0.05). CPT, continuous performance test, SDMT, signal detection modalities test; SDT, signal detection test.

### 3.2 The effect of schoolyard greening on social behavior, emotion recognition and attentional bias

**Figure 1:**
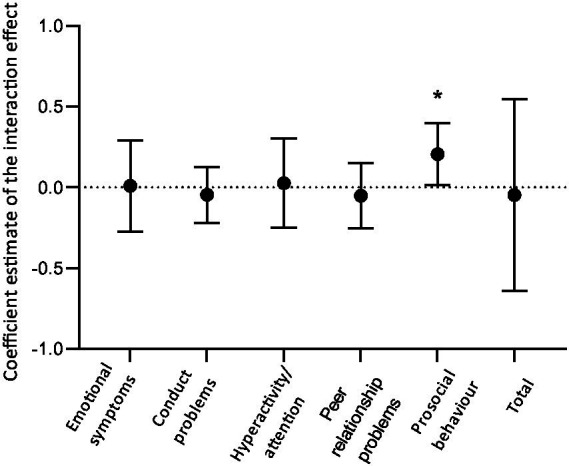
Estimates of the interaction effect involving group and examination number for the results of the SDQ. The model was adjusted for age, sex, country, education level of the household, the capacity to manage household incomes, and season. Error bars represent 95% CI. *p < 0.05.

The intervention group showed improved social behavior throughout greening compared to the control group, based on the mixed-effect models. A positive significant association was found for the interaction effect between intervention and control group throughout greening on prosocial behavior over time (*β* = 0.21, 95% CI 0.01–0.40, *p* = 0.03, Figure 1), after adjustment for the following covariates: age, sex, country in which the school is located, education level of the household, the capacity to manage household incomes, and season. No significant intervention effects over time were found for emotional problems, conduct problems, hyperactivity/inattention, peer relationship problems, and the total difficulty score. Over the course of the examinations, the intervention group showed a trend of an increase in the proportion of first fixation duration to the eyes as compared to the forehead and mouth (*β* = 0.03, 95% CI −0.001 to 0.05, *p* = 0.06). No effect of greening was shown on the proportion of the first fixation duration to the emotion happy as compared to the neutral face and angry and fearful emotions (*β* = −0.004, 95% CI −0.02 to 0.02, *p* = 0.67).

## 4 Discussion

This 2-year intervention study indicated a significant improvement in prosocial behavior during greening in the intervention schools compared to control schools, as assessed through a validated questionnaire, along with a potential attentional bias towards the eyes during facial recognition in eye-tracking assessments. Additionally, greening in the intervention schools was associated with enhanced cognitive processes, including improved reaction times in a selective attention test and a significant increase in pupil diameter during two cognitive tests, suggesting improved engagement or focus in children (46).

Our findings on prosocial behavior align with observational studies that associate the (perceived) quality of nearby green spaces with increased prosocial behavior (55, 56). Spending time in green schoolyards can increase social interactions, which may help with developing prosocial behavior (23, 36, 57–59). Richardson et al. (60) found a statistically significant association between neighborhood green space and prosocial behavior among children. Research emphasizes that prosocial behavior contributes significantly to various aspects of youth development. Encouraging prosocial behavior among children has been linked to improvements in academic performance (61, 62), enhanced social competence (63), and strengthened problem-solving abilities (64). In contrast to these studies, Balseviciene et al. (65) found a negative association between residential green space and prosocial behavior. Van Dijk-Wesselius et al. (36) has found a significant decrease in prosocial behavior after schoolyard greening in grade 6, while they found a significant increase in grades 4–6. Other studies report no statistically significant association between green exposure and prosocial behavior (66, 67). In general, a rise in prosocial behavior often indicates a healthier, more supportive social environment, which in turn is beneficial for overall wellbeing (68). Furthermore, the observed attentional bias towards the eyes in the facial recognition tasks could suggest improved prosocial behavior, given that eye contact is important for social development (69–72).

This study demonstrated that greening schools may improve cognitive performance. In addition to the restorative effects of nature on attention, exposure to green environments encourages children to be more physically active, which may pose a possible mechanism for improved cognitive performance (15, 16, 73, 74). We used cognitive tests as a proxy for cognitive performance and measured the pupil diameter as an indicator of the ability to focus. Several studies with interventional and observational design are in agreement with our results on cognition (8, 13, 36, 42). An intervention study in the Netherlands found that adding more greenery to schoolyards can positively improve children’s attention after breaks and their social wellbeing (36). Additionally, an observational study involving 2,593 children in Barcelona found an association between residential exposure to green space and enhanced cognitive development (13). Likewise, observational research by Bijnens et al. (8) revealed that higher percentages of residential green space are associated with greater intelligence and fewer behavioral problems in children aged 7–15 living in urban settings. Consistent with our results, an observational Belgian study involving 307 primary schoolchildren found that exposure to residential surrounding green space was associated with better selective attention performance based on the Stroop test (42). A significant increase in pupil diameter during the Spanne and Signal Detection Test was observed for the intervention group during greening, compared to the control group, while a trend was observed for the Signal Detection Modalities test and Stroop test. These findings suggest that the increased pupil diameter during the cognitive tasks may indicate heightened engagement or focus of children in a greening environment (75, 76). This is also in line with the Attention Restoration Theory, suggesting the positive impact of green infrastructure on cognitive restoration (16). However, further research is needed to explore the long-term implications of pupil diameter changes, particularly in relation to sustained cognition. Growing evidence shows that exposure to green environments can shape cognitive development and behavior from an early age, which indicates the importance of implementing green space from a young age (77–80). Dadvand et al. (77) found that long-term greenness exposure early in life is associated with beneficial structural changes in the brain. Similarly, Liao et al. (78) reported that neighborhood greenspace correlates with better neurodevelopment among children aged two and younger. Further, evidence gathered by Mason et al. (81) suggests that even brief passive exposure to nature supports improvements in attention and working memory across primary, secondary, and tertiary education levels.

As for the hypotheses specifically related to emotional problems, conduct problems, hyperactivity/inattention, peer relationship problems, or the total difficulty score, no significant effects were detected. Several factors may explain these findings. First, these behavioral domains could be less sensitive to environmental changes over shorter time frames, especially when baseline levels are already within the normal range. Second, the relatively small sample size and the variability inherent to the parent-reported questionnaire may have limited statistical power to detect subtle changes in these outcomes. Findings of this study highlight the importance of introducing greenery into environments such as schoolyards from a young age, which may have lasting positive effects on children’s cognitive development. However, further research, including a bigger sample size, is necessary to confirm our positive results.

An important difference between the approach of other studies and our study is that greening was specifically designed to improve biodiversity (23, 35, 36). ‘The Biodiversity Hypothesis’ proposes that exposure to a rich diversity of microorganisms in natural environments contributes to human health by supporting the development and regulation of the immune system, reducing inflammation, and promoting overall wellbeing (82). Although we were unable to specifically measure the biodiversity impacts of greening, the intervention in our study may have led to higher exposure to microbial diversity. Exposure to biodiversity has been linked to various benefits for mental health and cognitive development among children (29). Using green land cover as a measure of biodiversity, Maes et al. (83) found that increased natural spaces and woodlands were positively associated with improved executive functioning and higher total SDQ scores. In addition, findings by van Dijk-Wesselius et al. (36) showed greater appreciation of the schoolyard after schoolyard greening among young children and, in particular, girls.

Our intervention study had a number of strengths. The study incorporates a design with control groups and the conduction of multiple follow-up measurements. During the study, cognitive tests were used as an indicator of cognitive performance. Repeated testing of cognitive functioning leads to improved results due to a learning curve (84). However, this learning effect due to repeated measures should be the same in both groups (intervention versus control). This study has also some limitations. Schools were selected based on willingness to participate, which may have led to selection bias. However, no significant baseline differences were observed between the intervention and control schools. Including both intervention and control schools from Belgium and the Netherlands helped mitigate potential regional biases and may enhance the generalizability of these findings. Due to the explorative nature of this study, no sample size calculations were performed and the sample size was limited. Another limitation is that we are unable to quantify the exact extent of biodiversity enrichment in the intervention schools. Nevertheless, while this information would provide additional context for the study, the analyses are based on doing the intervention, and this availability would not affect the statistical analyses or the results. Future research should focus on larger sample sizes and extended follow-up periods to more effectively assess the potential long-term benefits of schoolyard greening.

This project provides promising results for future research. In summary, this study’s findings suggest that the intervention may foster improvements in prosocial behavior and enhance selective attention. These results warrant further investigation with larger studies. Our findings might have important implications for enhancing cognitive performance and prosocial wellbeing among primary schoolchildren, which shows the importance of introducing a biodiverse nature from a young age.

## Data Availability

The datasets used and/or analysed during the current study are available from the corresponding author on reasonable request.
