# Supplementary material for: The impact of greening interventions in school grounds on social behavior and cognitive performance among primary school children
Source: Front Public Health. 2025 Oct 29;13:1620199. doi: 10.3389/fpubh.2025.1620199 (PMC12605029; doi:10.3389/fpubh.2025.1620199)
Supplement: Supplementary file 1 [file Data_Sheet_1.pdf]

SUPPLEMENTARY

Supplementary Table 1: Demographic characteristics of participating children per school.

	Herx (n = 37)	de Beerring 2 (n = 52)	Loedoes (n = 49)	de Liaan (n = 31)
	**Mean ± SD or n (%)**	**Mean ± SD or n (%)**	**Mean ± SD or n (%)**	**Mean ± SD or n (%)**
**Square meters schoolyard per child**	25.9 m²	27.0 m²	10.0 m²	11.7 m²
**Estimated % greened area after the intervention**	19.8%	0%	19.6%	0%
**Age, years**	10.6 (0.840)	10.7 (0.951)	9.62 (0.930)	9.53 (1.00)
**Sex**				
Boy	12 (32.4%)	25 (48.1%)	25 (51.0%)	14 (45.2%)
Girl	25 (67.6%)	27 (51.9%)	24 (49.0%)	17 (54.8%)
**BMI Z-score**	0.329 (1.33)	0.533 (1.30)	0.264 (1.02)	0.484 (0.729)
**Education mother**				
Low	20 (54.1%)	14 (26.9%)	0 (0%)	0 (0%)
Middle	10 (27.0%)	23 (44.2%)	17 (34.7%)	11 (35.5%)
High	6 (16.2%)	13 (25.0%)	18 (36.7%)	14 (45.2%)
Missing	1 (2.7%)	2 (3.8%)	14 (28.6%)	6 (19.4%)
**Education father**				
Low	8 (21.6%)	2 (3.8%)	1 (2.0%)	0 (0%)
Middle	17 (45.9%)	25 (48.1%)	11 (22.4%)	10 (32.3%)
High	11 (29.7%)	20 (38.5%)	22 (44.9%)	13 (41.9%)
Missing	1 (2.7%)	5 (9.6%)	15 (30.6%)	8 (25.8%)
**Income**				
Very difficult	2 (5.4%)	1 (1.9%)	0 (0%)	0 (0%)
Difficult	4 (10.8%)	2 (3.8%)	0 (0%)	1 (3.2%)
Average	16 (43.2%)	23 (44.2%)	5 (10.2%)	4 (12.9%)
Rather easy	8 (21.6%)	19 (36.5%)	3 (6.1%)	3 (9.7%)
Very easy	6 (16.2%)	7 (13.5%)	3 (6.1%)	7 (22.6%)
Missing	1 (2.7%)	0 (0%)	38 (77.6%)	16 (51.6%)

This study employed a non-randomized controlled trial involving four primary schools, with one intervention (Herx, Herk-De-Stad) and one control school (De Beerring 2, Beringen) located in Limburg, Belgium, and the other intervention (Loedoes, Sittard) and control (de Liaan, Helden) schools located in Limburg, the Netherlands. General characteristics were collected regarding information about the birth date of the child, the sex of the child, parental education, and income using a questionnaire before the baseline examination.
Values are presented as mean ± standard deviation (SD) for continuous variables and n (%) for categorical variables. Values regarding the total area of the schoolyard per child and the percentage greening of the schoolyard only provide an estimated value.

Supplementary Table 2: Overview of questionnaires and measurements for data collection measures within the study.

Data collection activity	Parents	Children	Time point
**General questionnaire**			
Personal data (birth date, sex, ethnicity, addresses, parity, SES, education level of the parents)	x		baseline
Household smoking habits	x		baseline, B, C, D, E
**Cognitive performance and pupil diameter**			
Battery of cognitive tasks		x	baseline, B, C, D, E
Pupil diameter based on eye tracking during cognitive tasks		x	baseline, B, C, D, E
**Social behavior, attentional bias and Emotion recognition**			
Strengths and Difficulties Questionnaire (SDQ)	x		baseline, B, C, D, E
Attentional bias		x	baseline, B, C, D, E
Emotion recognition		x	baseline, B, C, D, E

This table lists all data collection activities for parents and children at each study time point in the two-year follow-up of this study, assessing the impact of schoolyard greening on children's cognitive and socio-emotional outcomes. Activities include: a general questionnaire filled out by the parents at home (personal data, household smoking habits, parental education, socio-economic status), cognitive performance tests, pupil diameter measurement via eye-tracking, the Strengths and Difficulties Questionnaire (SDQ) for behaviour, and eye-tracking tasks for attentional bias and emotion recognition. Data collection took place at five time points: baseline (November 2021), follow-up B (May 2022), follow-up C (November 2022), follow-up D (May 2023), and follow-up E (November 2023). Due to practical reasons, examinations B and E were not conducted in Dutch primary schools.

Supplementary material 1: Example of AOIs that were set using the Tobii Pro software.

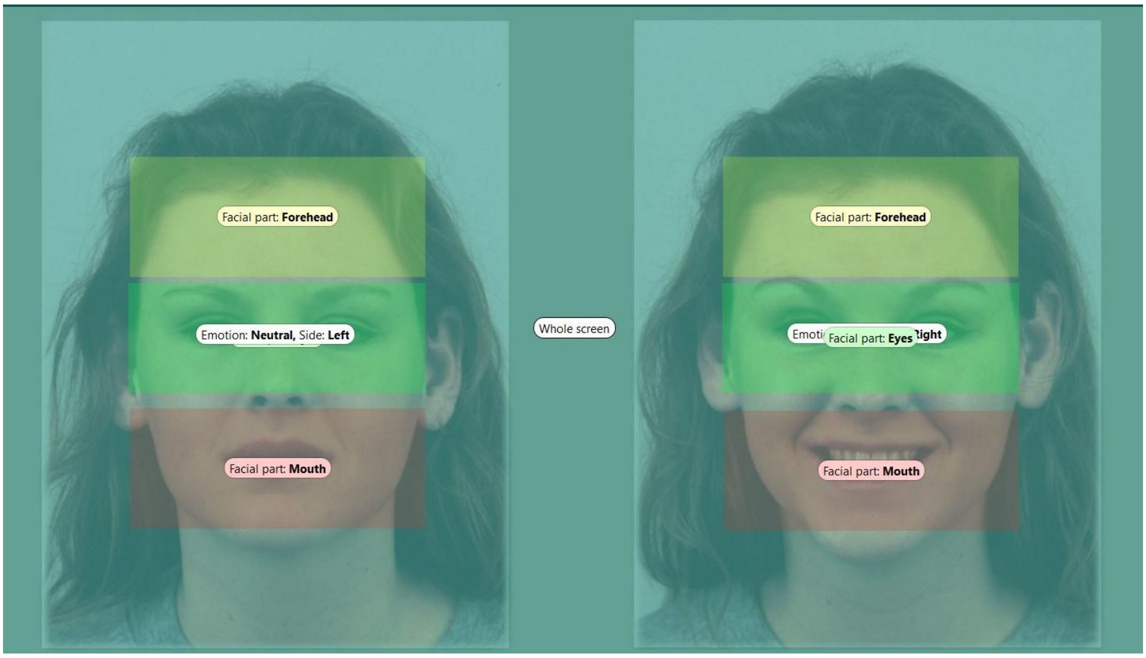

Supplementary Table 3: Baseline comparison of the outcome measures between the control and intervention schools.

Outcome	Test statistic (df)	p-value
**Cognitive tests**		
CPT	0.19 (151.37)	0.85
SDMT	-0.45 (155.92)	0.65
SPANNE forward	-0.1 (152.9)	0.92
SPANNE backward	1.4 (152.17)	0.16
SDT	-0.99 (107.15)	0.32
STROOP	-0.01 (149.56)	0.99
**Pupil diameter for assessing mental effort**		
Mean pupil diameter CPT	-0.67 (84.66)	0.50
Mean pupil diameter SDMT	1.64 (88.31)	0.10
Mean pupil diameter SPANNE	1.64 (88.15)	0.10
Mean pupil diameter SDT	1.74 (86.64)	0.09
Mean pupil diameter STROOP	0.15 (79.8)	0.88
**Social behaviour based on SDQ**		
Emotional problems	0.12 (126.49)	0.91
Conduct problems	-1.8 (134.88)	0.07
Hyperactivity/inattention	-1.56 (125.62)	0.12
Peer relationship problems	-0.97 (131.67)	0.34
Prosocial behaviour	1.26 (117.69)	0.21
Total difficulty score	-1.36 (126.48)	0.18
**Emotion recognition**		
Duration of first fixation proportion eyes	0.37 (84.47)	0.72
**Attentional bias**		
Duration of first fixation proportion happy	-1.13 (80.2)	0.26

Group comparisons for continuous variables were conducted using Welch's two-sample t-test. The p-value for the difference between groups is reported. df = degrees of freedom. p-values < 0.05 are considered statistically significant. Abbreviations: CPT; Continuous Performance Test, SDMT; Signal Detection Modalities test, SDT; Signal Detection test.

Supplementary Table 4: Estimated means and standard errors of outcome measures of the mixed effects models in the control and intervention groups for all measurements.

	Baseline		Follow-up B		Follow-up C		Follow-up D		Follow-up E	
**Outcome measure**	**Control**	**Intervention**	**Control**	**Intervention**	**Control**	**Intervention**	**Control**	**Intervention**	**Control**	**Intervention**
**Emotional problems**	1.69 (0.52)	1.88 (0.53)	1.68 (0.53)	2.34 (0.58)	1.70 (0.52)	2.48 (0.51)	1.58 (0.56)	1.75 (0.51)	1.10 (0.78)	1.38 (0.76)
**Conduct problems**	0.85 (0.36)	1.36 (0.36)	0.87 (0.36)	1.51 (0.38)	0.68 (0.35)	1.43 (0.34)	0.57 (0.37)	0.94 (0.34)	0.59 (0.51)	0.81 (0.49)
**Hyperactivity/inattention**	2.94 (0.61)	3.26 (0.62)	3.30 (0.61)	3.64 (0.65)	3.29 (0.60)	3.57 (0.58)	3.20 (0.64)	3.44 (0.59)	3.42 (0.85)	4.36 (0.82)
**Peer relationship problems**	1.17 (0.35)	1.66 (0.36)	1.04 (0.36)	1.05 (0.40)	1.36 (0.35)	1.75 (0.35)	1.52 (0.38)	1.66 (0.35)	1.35 (0.54)	1.89 (0.53)
**Prosocial behaviour**	8.52 (0.42)	7.93 (0.42)	8.48 (0.42)	8.15 (0.44)	8.62 (0.41)	8.60 (0.40)	8.73 (0.43)	8.66 (0.40)	8.49 (0.58)	8.90 (0.56)
**Total difficulty score**	6.73 (1.42)	8.20 (1.43)	6.97 (1.41)	8.50 (1.48)	7.06 (1.40)	9.28 (1.35)	6.93 (1.48)	7.82 (1.36)	6.39 (1.92)	8.55 (1.85)
**Duration of first fixation proportion eyes**	0.72 (0.05)	0.67 (0.05)	0.70 (0.04)	0.66 (0.04)	0.68 (0.05)	0.67 (0.04)	0.67 (0.05)	0.70 (0.04)	0.61 (0.07)	0.68 (0.07)
**Duration of first fixation proportion happy**	0.33 (0.02)	0.33 (0.02)	0.33 (0.02)	0.35 (0.02)	0.33 (0.02)	0.33 (0.02)	0.35 (0.03)	0.35 (0.02)	0.33 (0.04)	0.31 (0.05)
**CPT**	509.14 (17.94)	491.07 (18.25)	509.53 (18.53)	486.00 (18.87)	512.01 (18.52)	501.63 (18.04)	505.66 (19.40)	491.48 (18.97)	560.37 (31.14)	500.69 (29.39)
**SDMT**	4.07 (0.20)	4.02 (0.21)	3.83 (0.21)	3.62 (0.21)	3.72 (0.21)	3.59 (0.20)	3.83 (0.22)	3.58 (0.21)	3.71 (0.32)	3.84 (0.37)
**SPANNE forward**	4.11 (0.20)	4.06 (0.20)	4.43 (0.21)	4.26 (0.21)	4.34 (0.21)	4.15 (0.20)	4.28 (0.21)	4.19 (0.20)	4.39 (0.32)	4.09 (0.33)
**SPANNE backward**	3.19 (0.23)	2.99 (0.24)	3.53 (0.24)	3.51 (0.24)	3.45 (0.24)	3.23 (0.23)	3.71 (0.24)	3.29 (0.23)	3.16 (0.35)	3.22 (0.36)
**SDT**	1263.93 (79.29)	1339.36 (82.79)	1265.94 (83.39)	1299.67 (86.41)	1283.49 (82.66)	1356.56 (79.44)	1285.59 (85.60)	1347.79 (80.83)	1405.60 (127.46)	965.35 (134.20)
**STROOP**	3.26 (0.02)	3.25 (0.02)	3.24 (0.02)	3.20 (0.02)	3.24 (0.02)	3.22 (0.02)	3.25 (0.02)	3.22 (0.02)	3.29 (0.03)	3.13 (0.03)
**Mean pupil diameter CPT**	3.39 (0.12)	3.46 (0.12)	3.52 (0.12)	3.00 (0.13)	3.27 (0.13)	3.32 (0.11)	3.15 (0.13)	3.30 (0.13)	3.09 (0.19)	3.28 (0.19)
**Mean pupil diameter SDMT**	4.26 (0.13)	3.99 (0.13)	3.95 (0.13)	3.52 (0.14)	4.00 (0.14)	3.94 (0.13)	3.71 (0.15)	3.65 (0.14)	3.79 (0.22)	3.89 (0.23)
**Mean pupil diameter SPANNE**	3.96 (0.12)	3.71 (0.12)	3.87 (0.12)	3.29 (0.13)	3.78 (0.13)	3.68 (0.12)	3.46 (0.13)	3.46 (0.13)	3.43 (0.20)	3.67 (0.21)
**Mean pupil diameter SDT**	4.09 (0.13)	3.84 (0.14)	3.92 (0.13)	3.35 (0.14)	3.84 (0.14)	3.76 (0.13)	3.53 (0.15)	3.51 (0.14)	3.50 (0.22)	3.74 (0.23)
**Mean pupil diameter STROOP**	3.77 (0.13)	3.70 (0.13)	3.77 (0.13)	3.32 (0.14)	3.60 (0.14)	3.65 (0.13)	3.41 (0.15)	3.43 (0.14)	3.38 (0.21)	3.73 (0.22)

Abbreviations: CPT; Continuous Performance Test, SDMT; Signal Detection Modalities test, SDT; Signal Detection test

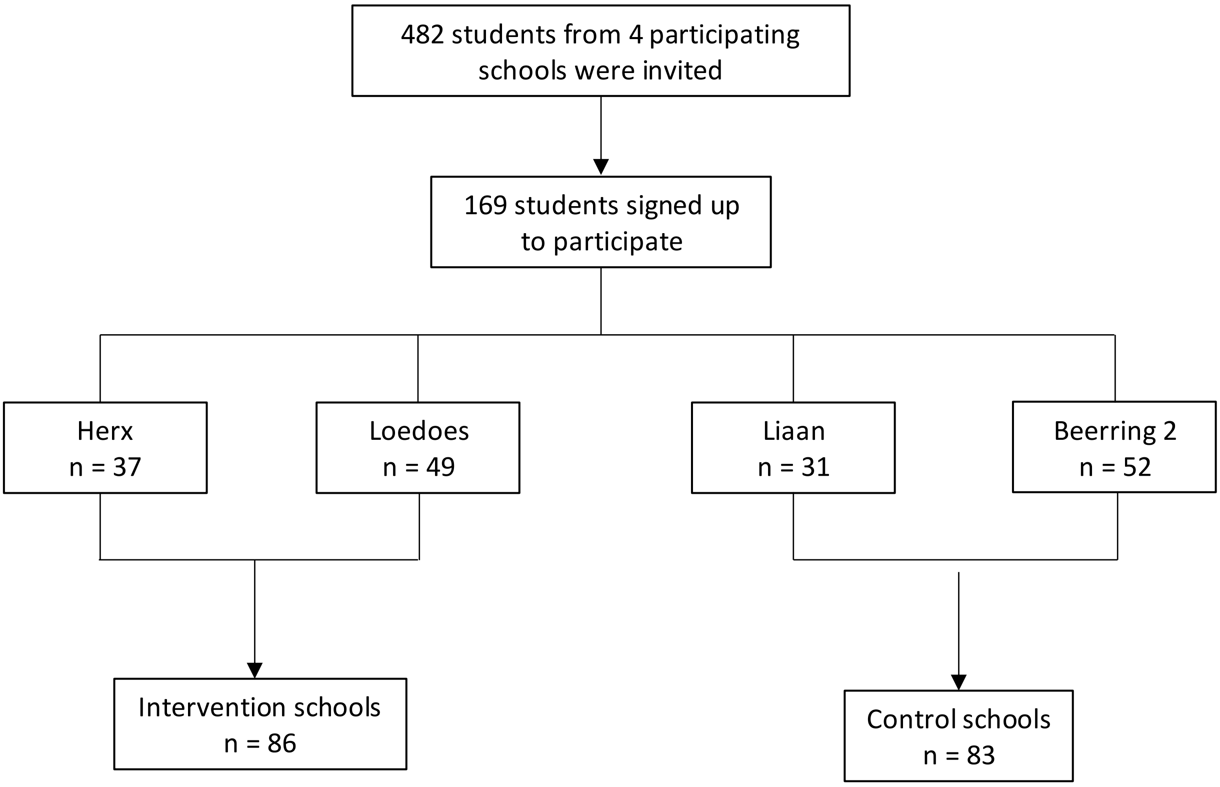

Supplementary Figure 1: Flowchart describing included participants
